# Author Correction: Cooperation of Striatin 3 and MAP4K4 promotes growth and tissue invasion

**DOI:** 10.1038/s42003-022-03855-2

**Published:** 2022-08-26

**Authors:** Jessica Migliavacca, Buket Züllig, Charles Capdeville, Michael A. Grotzer, Martin Baumgartner

**Affiliations:** 1 Pediatric Molecular Neuro-Oncology Research, Division of Oncology, Children’s Research Center, University Children’s Hospital Zürich, Zürich, Switzerland; 2 Division of Oncology, University Children’s Hospital Zürich, Zürich, Switzerland

**Keywords:** Growth factor signalling, Paediatric cancer, Cancer models, Cell invasion

Correction to: *Communications Biology* 10.1038/s42003-022-03708-y, published online 08 August 2022.

The original version of the Supplementary information associated with this Article was missing Supplementary tables 1–4 and the original immunoblot images. This information has now been added to the Supplementary information file and corrected in the PDF and HTML versions of the Article.

## Supplementary information

Updated supplementary file

