# Supplementary material for: Cooperation of Striatin 3 and MAP4K4 promotes growth and tissue invasion
Source: Commun Biol. 2022 Aug 8;5:795. doi: 10.1038/s42003-022-03708-y (PMC9360036; doi:10.1038/s42003-022-03708-y)
Supplement: Supplementary file 3 — Description of Additional Supplementary Files [file 42003_2022_3708_MOESM3_ESM.pdf]

Description of Additional Supplementary Files

File name: Supplementary information
Description: Antibodies and primers used, uncropped immunoblots
File Name: Supplementary data 1
Description: MAP4K4 interactomes in HEK-293T cells
File Name: Supplementary data 2
Description: MAP4K4 interactomes in DAOY cells
File Name: Supplementary data 3
Description: Log fold change (LFC) and P values for peptide phosphory
File Name: Supplementary data 4
Description: IFA metadata
File name: Supplementary data 5.xlsx
Description: Source data Figure 1
File name: Supplementary data 6.xlsx
Description: Source data Figure 2
File name: Supplementary data 7.xlsx
Description: Source data Figure 3
File name: Supplementary data 8.xlsx
Description: Source data Figure 4
File name: Supplementary data 9.xlsx
Description: Source data Figure 5
File name: Supplementary data 10.xlsx
Description: Source data Figure 6
File name: Supplementary data 11.xlsx
Description: Source data Figure S2
File name: Supplementary data 12.xlsx
Description: Source data Figure S3
File name: Supplementary data 13.xlsx
Description: Source data Figure S4
File name: Supplementary data 14.xlsx
Description: Source data Figure S6
File name: Supplementary data 15.xlsx
Description: Source data Figure S7
File name: Supplementary data 16.xlsx
Description: Source data Figure S8
File name: Supplementary data 17.xlsx
Description: Source data Figure S9
