# Supplementary material for: Cooperation of Striatin 3 and MAP4K4 promotes growth and tissue invasion
Source: Commun Biol. 2022 Aug 8;5:795. doi: 10.1038/s42003-022-03708-y (PMC9360036; doi:10.1038/s42003-022-03708-y)
Supplement: Supplementary file 21 — Reporting Summary [file 42003_2022_3708_MOESM21_ESM.pdf]

nature portfolio

Corresponding author(s): Martin Baumgartner

Last updated by author(s): May 23, 2022

Reporting Summary

Nature Portfolio wishes to improve the reproducibility of the work that we publish. This form provides structure for consistency and transparency in reporting. For further information on Nature Portfolio policies, see our Editorial Policies and the Editorial Policy Checklist.

Statistics

For all statistical analyses, confirm that the following items are present in the figure legend, table legend, main text, or Methods section.

n/a	Confirmed	
☐	☒	The exact sample size (n) for each experimental group/condition, given as a discrete number and unit of measurement
☐	☒	A statement on whether measurements were taken from distinct samples or whether the same sample was measured repeatedly
☐	☒	The statistical test(s) used AND whether they are one- or two-sided<br>*Only common tests should be described solely by name; describe more complex techniques in the Methods section.*
☐	☒	A description of all covariates tested
☐	☒	A description of any assumptions or corrections, such as tests of normality and adjustment for multiple comparisons
☐	☒	A full description of the statistical parameters including central tendency (e.g. means) or other basic estimates (e.g. regression coefficient) AND variation (e.g. standard deviation) or associated estimates of uncertainty (e.g. confidence intervals)
☐	☒	For null hypothesis testing, the test statistic (e.g. F, t, r) with confidence intervals, effect sizes, degrees of freedom and P value noted<br>*Give P values as exact values whenever suitable.*
☒	☐	For Bayesian analysis, information on the choice of priors and Markov chain Monte Carlo settings
☒	☐	For hierarchical and complex designs, identification of the appropriate level for tests and full reporting of outcomes
☒	☐	Estimates of effect sizes (e.g. Cohen's d, Pearson's r), indicating how they were calculated

Our web collection on statistics for biologists contains articles on many of the points above.

Software and code

Policy information about availability of computer code

Data collection	BioRad Image Lab 5.2.1. was used for chemiluminescence detection of Western blots.<br>Leica SP8 confocal microscope, Zeiss AxioObserver 2 mot plus fluorescence microscope or PerkinElmer Operetta high-content imaging system were used for immunofluorescence microscopy.<br>Thermos scientific Orbitrap Fusion Tribrid Mass Spectrometry was used for measurement in BioID experiments.<br>ABI 7900UT Fast Real-Time PCR System was used for RT-qPCR data acquisition.<br>BioTek Cytation 3 plate reader was used for measurement of colorimetric assays (cellTox green).<br>PamGene PamStation®12 was used to process the PamChip® microarrays for protein kinase activity profiling.
Data analysis	GraphPad Prism 8 software was used for statistical analysis.<br>ImageJ (National Institutes of Health, USA) software was to quantify the integrated density of immuno-reactive bands of immunoblot images, to quantify the area of the colonies in the colony formation assays, and to quantify the fluorescence microscope images of the ex vivo organotypic cerebellum slice cultures.<br>Protein identification and quantification of BioID experiments were performed using Scaffold (version 4.8.9, Proteome Software Inc., Portland, OR).<br>Gene ontology enrichment analysis was performed using Metascape (http://metascape.org/).<br>Harmony 4.5 software (PerkinElmer) was used to quantify the sum of the distance invaded by the cells from the center of the spheroid in the spheroid invasion assay.<br>For the protein kinase activity profiling, the fluorescent signal intensity for each peptide on the PamChip was quantitated by BioNavigator software (PamGene). An upstream kinase prediction tool based on the PamApp on BioNavigator Analysis software was used to generate a putative list of kinases responsible for phosphorylating the phosphosites on the PamChip.<br>Venn diagram analyses were performed in Venny 2.1 (https://bioinfogp.cnb.csic.es/tools/venny/).

To group the kinases into sequence families, phylogenetic trees were created using the web-based kinome tool CORAL (http://phanstiel-lab.med.unc.edu/CORAL/).

For manuscripts utilizing custom algorithms or software that are central to the research but not yet described in published literature, software must be made available to editors and reviewers. We strongly encourage code deposition in a community repository (e.g. GitHub). See the Nature Portfolio guidelines for submitting code & software for further information.

Data

Policy information about availability of data

All manuscripts must include a data availability statement. This statement should provide the following information, where applicable:

- Accession codes, unique identifiers, or web links for publicly available datasets
- A description of any restrictions on data availability
- For clinical datasets or third party data, please ensure that the statement adheres to our policy

Mass spectrometry proteomic data of BioID experiments have been deposited to the ProteomeXchange Consortium via the PRIDE partner repository with the data set identifier PXD031863 and 10.6019/PXD031863 for the MAP4K4 interactome in HEK293T cells (Table S1) and PXD031870 and 10.6019/PXD031870 for the MAP4K4 interactome in DAOY cells (Table S2).

Human research participants

Policy information about studies involving human research participants and Sex and Gender in Research.

Reporting on sex and gender	*Use the terms sex (biological attribute) and gender (shaped by social and cultural circumstances) carefully in order to avoid confusing both terms. Indicate if findings apply to only one sex or gender; describe whether sex and gender were considered in study design whether sex and/or gender was determined based on self-reporting or assigned and methods used. Provide in the source data disaggregated sex and gender data where this information has been collected, and consent has been obtained for sharing of individual-level data; provide overall numbers in this Reporting Summary. Please state if this information has not been collected. Report sex- and gender-based analyses where performed, justify reasons for lack of sex- and gender-based analysis.*
Population characteristics	*Describe the covariate-relevant population characteristics of the human research participants (e.g. age, genotypic information, past and current diagnosis and treatment categories). If you filled out the behavioural & social sciences study design questions and have nothing to add here, write "See above."*
Recruitment	*Describe how participants were recruited. Outline any potential self-selection bias or other biases that may be present and how these are likely to impact results.*
Ethics oversight	*Identify the organization(s) that approved the study protocol.*

Note that full information on the approval of the study protocol must also be provided in the manuscript.

Field-specific reporting

Please select the one below that is the best fit for your research. If you are not sure, read the appropriate sections before making your selection.

☒ Life sciences ☐ Behavioural & social sciences ☐ Ecological, evolutionary & environmental sciences

For a reference copy of the document with all sections, see nature.com/documents/nr-reporting-summary-flat.pdf

Life sciences study design

All studies must disclose on these points even when the disclosure is negative.

Sample size	No statistical method for sample size calculations were performed. We have used minimal sample size sufficient to detect biological differences. Numbers of samples are indicated in the figure legends for each panel.
Data exclusions	Outliers found in technical replicates of Ct-threshold measurements within RT-qPCR experiments (pre-established SD > 0.5) were removed from the analysis.<br>Otherwise no data was excluded.
Replication	The experiments were performed in at least three independent biological replicates (n=3) unless differently indicated. Number of biological replicates are given where applicable.
Randomization	Our experiments were not randomized, because we have controls run side-by-side with the experimental samples.
Blinding	Our experiments were not blinded, because we have controls run side-by-side with the experimental samples.

Reporting for specific materials, systems and methods

We require information from authors about some types of materials, experimental systems and methods used in many studies. Here, indicate whether each material, system or method listed is relevant to your study. If you are not sure if a list item applies to your research, read the appropriate section before selecting a response.

Materials & experimental systems

n/a	Involved in the study
☐	☒ Antibodies
☐	☒ Eukaryotic cell lines
☒	☐ Palaeontology and archaeology
☐	☒ Animals and other organisms
☒	☐ Clinical data
☒	☐ Dual use research of concern

Methods

n/a	Involved in the study
☒	☐ ChIP-seq
☒	☐ Flow cytometry
☒	☐ MRI-based neuroimaging

Antibodies

Antibodies used

Antibodies Used for Immune-Blotting

- Anti-β-tubulin - clone TUB 2.1 - Sigma Aldrich (#T5201) - 1:1000 dilution
- Anti-GAPDH - clone 14C10 - Cell signaling technologies (#2118S) - 1:2000 dilution
- Anti-FLAG® M2 - clone M2 - Sigma Aldrich (#F1804) - 1:1500 dilution
- Streptavidin HRP - Jackson Immunoresearch (#16-030-084) - 1:500 dilution
- Anti-MAP4K4 - Abcam (#ab80418) - 1:2000 dilution
- Anti-STRN4 - Abcam (#ab194948) - 1:2000 dilution
- Anti-STRN3 - clone S68 - Thermo Fisher Scientific (#MA1-46461) - 1:2000 dilution
- Anti-STRIP1/FAM40A - Abcam (#ab199851) - 1:2000 dilution
- Anti-PKCθ - clone E1I7Y - Cell signaling technologies (#13643) - 1:1000 dilution
- Anti-phospho-PKC θ Thr538 - Cell signaling technologies (#9377) - 1:1000 dilution
- Anti-VASP - clone 9A2 - Cell signaling technologies (#3132) - 1:1000 dilution
- Anti-phospho-VASP (Ser157) - Abcam (#ab47268) - 1:750 dilution
- Anti-mouse HRP linked - Cell signaling technologies (#7076) - 1:5000 dilution
- Anti-rabbit HRP linked - Cell signaling technologies (#7074) - 1:5000 dilution
- EasyBlot Anti-mouse HRP - GeneTex (#GTX221667-01) - 1:1000 dilution

Antibodies Used for Immune-Precipitation

- Anti-FLAG® M2 - clone M2 - Sigma Aldrich (#F1804) - 3 µg/mg lysate
- Anti-STRN4 - Abcam (#ab194948) - 3 µg/mg lysate
- Anti-STRIP1/FAM40A - Abcam (#ab199851) - 3 µg/mg lysate
- Normal rabbit IgG - Cell signaling technologies (#2729) - 3 µg/mg lysate

Antibodies/Dyes Used for Immune-Fluorescence

- Anti-BioID2 - clone SS QD1 - Novus Biologicals (#NBP2-59940) - 1:200 dilution
- Streptavidin Alexa Fluor 594 conjugate - Invitrogen (#S11227) - 1:500 dilution
- Anti-Calbindin - clone EP3478 - Abcam (#ab108404) - 1:1000 dilution
- Anti-GFAP - Abcam (#ab53554) - 1:250 dilution
- Anti-human nuclei - clone 3E1.3 - Millipore (#MAB4383) - 1:250 dilution
- Donkey Anti-Mouse IgG, Secondary Antibody, Alexa Fluor 488 - Thermo Fisher Scientific (#A32766) - 1:400 dilution
- Donkey Anti-Rabbit IgG, Cy3-conjugated - Jackson Immunoresearch (#711-165-152) - 1:250 dilution
- Donkey Anti-Goat IgG, Brilliant Violet 421 conjugated - Jackson Immunoresearch (#705-675-147) - 1:100 dilution
- Goat Anti-Mouse IgG, Secondary Antibody, Alexa Fluor 405 - Thermo Fisher Scientific (#A31553) - 1:200 dilution
- Hoechst - Sigma Aldrich (#B2883) - 1:2000 dilution

Validation

Validation for the following antibodies are available on the manufacturer's homepage

- Anti-β-tubulin - https://www.sigmaaldrich.com/CH/en/product/sigma/t5201
- Anti GAPDH - https://www.cellsignal.com/products/primary-antibodies/gapdh-14c10-rabbit-mab/2118
- Anti FLAG M2 - https://www.sigmaaldrich.com/CH/en/product/sigma/f1804
- Streptavidin HRP- https://www.jacksonimmuno.com/catalog/products/016-030-084
- Anti MAP4K4 - https://www.abcam.com/map4k4nik-antibody-ab80418.html
- Anti-STRN4 - https://www.abcam.com/striatin-4-antibody-ab194948.html
- Anti-STRN3 - https://www.thermofisher.com/antibody/product/STRN3-Antibody-clone-S68-Monoclonal/MA1-46461
- Anti-STRIP1/FAM40A - https://www.abcam.com/fam40a-antibody-ab199851.html

- Anti-PKCθ - https://www.cellsignal.com/products/primary-antibodies/pkcq-e1i7y-rabbit-mab/13643
- Anti-phospho-PKC θ - https://www.cellsignal.com/products/primary-antibodies/phospho-pkcq-thr538-antibody/9377
- Anti-VASP - https://www.cellsignal.com/products/primary-antibodies/vasp-9a2-rabbit-mab/3132
- Anti-phospho-VASP (Ser157) - https://www.cellsignal.com/products/primary-antibodies/vasp-9a2-rabbit-mab/3132
- Anti-mouse HRP - https://www.cellsignal.com/products/secondary-antibodies/anti-mouse-igg-hrp-linked-antibody/7076
- EasyBlot Anti-mouse HRP - https://www.genetex.com/Product/Detail/EasyBlot-anti-Mouse-IgG-HRP/GTX221667-01
- Anti-BioID2 - https://www.novusbio.com/products/a-aeolicus-bpl-bioid2-antibody-ss-qd1_nbp2-59940
- Streptavidin Alexa Fluor 594 conjugate - https://www.thermofisher.com/order/catalog/product/S11227
- Anti-Calbindin - https://www.abcam.com/calbindin-antibody-ep3478-ab108404.html
- Anti-GFAP - https://www.abcam.com/gfap-antibody-ab53554.html
- Anti-human nuclei - https://www.merckmillipore.com/CH/de/product/Anti-Nuclei-Antibody-clone-3E1.3,MM_NF-MAB4383
- Donkey Anti-Mouse IgG Secondary Antibody - https://www.thermofisher.com/antibody/product/Donkey-anti-Mouse-IgG-H-L-Highly-Cross-Adsorbed-Secondary-Antibody-Polyclonal/A32766
- Donkey Anti-Rabbit IgG, Cy3-conjugated - https://www.jacksonimmuno.com/catalog/products/711-165-152
- Donkey Anti-Goat IgG, Brilliant Violet 421 conjugated - https://www.jacksonimmuno.com/catalog/products/705-675-147
- Goat Anti-Mouse IgG, Secondary Antibody - https://www.thermofisher.com/antibody/product/Goat-anti-Mouse-IgG-H-L-Cross-Adsorbed-Secondary-Antibody-Polyclonal/A-31553
- Hoechst - https://www.sigmaaldrich.com/CH/en/product/sial/b2883

Eukaryotic cell lines

Policy information about cell lines and Sex and Gender in Research

Cell line source(s)	DAOY human MB cells were purchased from the American Type Culture Collection (ATCC, Rockville, MD, USA).<br>UW228 cells were generously provided by John Silber (Seattle, USA).<br>HD-MBO3 group 3 MB cells were generously provided by Till Milde (DKFZ, Germany).
Authentication	Cell line authentication and cross-contamination testing were performed by Multiplexion by single nucleotide polymorphism (SNP) profiling
Mycoplasma contamination	Cell lines were regularly tested for Mycoplasma and were Mycoplasma free.
Commonly misidentified lines<br>(See ICLAC register)	None as far as we know.

Animals and other research organisms

Policy information about studies involving animals; ARRIVE guidelines recommended for reporting animal research, and Sex and Gender in Research

Laboratory animals	Wild type C57BL/6JRj pregnant females were purchased from Janvier Labs.
Wild animals	No wild animals were used in this study.
Reporting on sex	*Indicate if findings apply to only one sex; describe whether sex was considered in study design, methods used for assigning sex. Provide data disaggregated for sex where this information has been collected in the source data as appropriate; provide overall numbers in this Reporting Summary. Please state if this information has not been collected. Report sex-based analyses where performed, justify reasons for lack of sex-based analysis.*
Field-collected samples	*For laboratory work with field-collected samples, describe all relevant parameters such as housing, maintenance, temperature, photoperiod and end-of-experiment protocol OR state that the study did not involve samples collected from the field.*
Ethics oversight	Mouse protocols for organotypic brain slice culture were approved by the Veterinary Office of the Canton Zürich (Approvals ZH134/17, ZH116/20).

Note that full information on the approval of the study protocol must also be provided in the manuscript.
